# Supplementary material for: Centralization or Equalization? Policy Trend Guidance for Improving Grain Production Security in China
Source: Foods. 2025 Mar 12;14(6):966. doi: 10.3390/foods14060966 (PMC11941064; doi:10.3390/foods14060966)
Supplement: Supplementary file 1 [file foods-14-00966-s001.zip › foods-3497719-supplementary.pdf]

Table S1. Grain production support policies issued at the Chinese government level and scores on different dimensions.

Serial Number	Date of issue	Typology	Title	Issuing authority	Link	Policy level score	Policy goal score	Policy measure score	Policy power score	Policy implementation score
1	1985/9/9	Notice	Notice to buy more grain in areas with good harvests	State Council	http://www.gov.cn/zhengce/content/2013-09/29/content_3534.htm	4	3	2	3	1
2	1985/11/5	Notice	Notice on the effective grasping of grain efforts	State Council	http://www.gov.cn/zhengce/content/2013-10/14/content_3522.htm	4	5	4	5	3
3	1986/1/17	Notice	Notice of the grain contract purchase mandate for 1986	State Council	http://www.gov.cn/zhengce/content/2013-09/29/content_3513.htm	4	5	4	5	3
4	1986/10/14	Notice	Notice on improving the grain contract purchasing system	State Council	http://www.gov.cn/zhengce/content/2013-10/22/content_3491.htm	4	5	4	3	5
5	1987/11/28	Notice	Notice on the current grain work	State Council	http://www.gov.cn/zhengce/content/2011-09/01/content_3437.htm	4	3	3	1	3
6	1988/9/27	Decision	Decision on strengthening grain management and stabilizing the grain market	State Council	https://www.gov.cn/zhengce/zhengceku/2011-08/31/content_3373.htm	4	4	4	5	3
7	1990/9/16	Decision	Decision on the establishment of a national specialized grain reserve system	State Council	http://www.gov.cn/zhengce/content/2013-10/22/content_3309.htm	4	4	3	5	1
8	1991/1/12	Notice	Notice of the state council on issues relating to the adjustment of grain purchase and marketing policies	State Council	http://www.gov.cn/zhengce/content/2016-07/28/content_5090217.htm	4	5	4	3	5
9	1994/4/9	Notice	Notice on strengthening the "grain basket" and grain, cotton and oil work	State Council	http://www.gov.cn/zhengce/content/2015-12/18/content_10445.ht	4	5	4	3	1

					m					
10	1994/5/9	Notice	Notice on deepening the reform of the grain purchasing and marketing system	State Council	http://www.gov.cn/zhengce/content/2016-07/28/content_5090562.htm	4	5	4	3	3
11	1995/4/2	Notice	Notice on deepening the reform of the grain, cotton and fertilizer purchasing and marketing system	State Council	http://www.gov.cn/zhengce/content/2018-04/13/content_5281683.htm	4	4	3	3	3
12	1995/6/10	Notice	Notice on the deepening of reforms in the grain sector to implement two lines of operation	State Council	http://www.gov.cn/zhengce/content/2018-04/17/content_5281685.htm	4	5	4	5	5
13	1996/5/9	Notice	Notice on adjustment of grain selling prices	State Council	http://www.gov.cn/zhengce/content/2017-09/11/content_5223198.htm	4	4	3	1	3
14	1996/11/10	Notice	Notice on the current work of grain purchase and storage	State Council	https://www.gov.cn/zhengce/content/202211/content_6604211.htm	4	4	3	3	1
15	1998/5/10	Decision	Decision on further deepening the reform of the grain circulation system	State Council	http://www.gov.cn/zhengce/content/2010-11/17/content_3190.htm	4	4	3	3	3
16	1998/5/19	Notice	Notice on improvements to the management of the grain risk fund	General Office of the State Council	http://www.gov.cn/zhengce/content/2010-11/17/content_1793.htm	3	5	4	1	3
17	1998/5/19	Opinion	Opinions on improving the grain price formation mechanism	General Office of the State Council	http://www.gov.cn/zhengce/content/2010-11/17/content_3183.htm	3	4	3	3	3
18	2000/2/2	Notice	Notice on some grain varieties withdrawn from the scope of the protection of the purchase price of the relevant issues	General Office of the State Council	http://www.gov.cn/gongbao/content/2000/content_60661.htm	3	4	3	3	3

19	2000/5/9	Notice	Notice on the Issuance of the Measures for Interprovincial grain Purchase Price Convergence	State Planning Commission	http://www.gov.cn/gongbao/content/2000/content_60520.htm	2	3	3	1	1
20	2000/6/10	Notice	Notice on Further Improving Policy Measures Related to grain Production and Circulation	State Council	http://www.gov.cn/gongbao/content/2000/content_60284.htm	4	4	4	3	1
21	2001/1/11	Opinion	Opinions on doing agriculture and rural work well in 2001	Central Committee of the Communist Party of China, State Council	http://www.gov.cn/gongbao/content/2001/content_61314.htm	4	3	3	1	3
22	2001/3/24	Notice	Notice on further improving the pilot work of rural tax and fee reforms	State Council	http://www.gov.cn/gongbao/content/2001/content_60763.htm	4	4	3	1	1
23	2001/7/31	Opinion	Opinions on further deepening the reform of the grain circulation system	State Council	http://www.gov.cn/gongbao/content/2001/content_60987.htm	4	3	2	1	3
24	2002/11/3	Notice	Notice on the issuance of the interim measures for the supervision and administration of the grain risk fund	Ministry of Finance, State Planning Commission, Food Bureau, Agricultural Development Bank	http://www.gov.cn/gongbao/content/2002/content_61697.htm	2	4	3	5	5
25	2003/3/27	Opinion	Opinions on comprehensively promoting the pilot reform of rural taxes and fees	State Council	http://www.gov.cn/gongbao/content/2003/content_62076.htm	4	4	4	3	1
26	2003/8/15	Regulation	Regulations on the management of central grain reserves	State Council	http://www.gov.cn/gongbao/content/2003/content_62392.htm	4	3	2	1	5

27	2003/12/31	Opinion	Opinions on a number of policies to promote increased incomes for farmers	Central Committee of the Communist Party of China, State Council	http://www.gov.cn/gongbao/content/2004/content_63144.htm	4	3	3	3	1
28	2004/2/20	Notice	Notice on the implementation of policy measures related to several policy opinions of the Central Committee of the Communist Party of China and the State Council on promoting the increase of farmers' income	General Office of the State Council	http://www.gov.cn/gongbao/content/2004/content_62695.htm	3	3	2	1	5
29	2004/5/23	Opinion	Opinions on further deepening the reform of the grain circulation system	State Council	http://www.gov.cn/gongbao/content/2004/content_62827.htm	4	4	4	1	5
30	2004/9/30	Notice	Notice on the establishment of drafting groups and thematic groups for guidance documents on the pilot work of deepening the rural tax and fee reforms	General Office of the State Council	http://www.gov.cn/gongbao/content/2004/content_63013.htm	3	2	2	1	3
31	2004/10/21	Decision	Decision on deepening reform and strict land management	State Council	http://www.gov.cn/gongbao/content/2004/content_63043.htm	4	4	3	5	3
32	2004/12/31	Opinion	Opinions on a number of policies to further strengthen rural work and enhance comprehensive agricultural production capacity	Central Committee of the Communist Party of China, State Council	http://www.gov.cn/gongbao/content/2005/content_63347.htm	4	4	4	3	1

33	2005/2/17	Notice	Notice on the implementation of policy measures related to several policy opinions of the Central Committee of the Communist Party of China and the State Council on further strengthening rural work and enhancing comprehensive agricultural production capacity	General Office of the State Council	http://www.gov.cn/gongbao/content/2005/content_63164.htm	3	3	2	1	5
34	2005/4/2	Notice	Notice of the State Council on the issuance of the work points for 2005	State Council	http://www.gov.cn/gongbao/content/2005/content_63217.htm	4	3	2	1	5
35	2005/4/4	Opinion	Opinions on deepening economic system reform in 2005	State Council	http://www.gov.cn/gongbao/content/2005/content_63235.htm	4	3	3	1	3
36	2005/5/26	Regulation	Regulations on the management of grain circulation	State Council	http://www.gov.cn/zwgk/2005-05/23/content_217.htm	4	4	4	3	3
37	2005/12/31	Notice	Opinions on promoting the construction of new socialist rural areas	Central Committee of the Communist Party of China, State Council	http://www.gov.cn/gongbao/content/2006/content_254151.htm	4	3	3	3	1
38	2006/2/10	Notice	Notice on the implementation of relevant policies and measures on several opinions of the Central Committee of the Communist Party of China and the State Council on promoting the construction of a new socialist	General Office of the State Council	http://www.gov.cn/gongbao/content/2006/content_245846.htm	3	2	2	1	5

			countryside						
39	2006/3/14	Outline	Outline of the eleventh five-year plan for national economic and social development of the People's Republic of China	National People's Congress	http://www.gov.cn/gongbao/content/2006/content_268766.htm	5	2	2	3	1
40	2006/3/19	Notice	Notice of the issuance of the main points of work for 2006	State Council	http://www.gov.cn/zhengce/content/2008-03/28/content_1767.htm	4	2	2	1	5
41	2006/5/13	Opinion	Opinions on improving the policy measures for the reform of the grain circulation system	State Council	https://www.gov.cn/zhengce/content/202211/content_6604183.htm	4	3	3	1	3
42	2006/12/31	Opinion	Opinions on actively developing modern agriculture and solidly promoting the construction of a new socialist countryside	Central Committee of the Communist Party of China, State Council	http://www.gov.cn/gongbao/content/2007/content_548921.htm	4	3	2	1	1
43	2007/2/10	Notice	Notice on the implementation of the relevant policies and measures of the Central Committee of the Communist Party of China and the State Council on actively developing modern agriculture and solidly promoting the construction of a new socialist	General Office of the State Council	http://www.gov.cn/gongbao/content/2007/content_564121.htm	3	2	2	1	5

			countryside							
44	2007/3/5	Report	Government working report	National People's Congress	http://www.gov.cn/gongbao/content/2007/content_595132.htm	5	2	3	1	1
45	2007/3/25	Notice	Notice of the issuance of the main points of work for 2007	State Council	http://www.gov.cn/gongbao/content/2007/content_609913.htm	4	3	3	1	5
46	2007/12/31	Opinion	Opinions on effectively strengthening agricultural infrastructure to further promote agricultural development and increase farmers' income	Central Committee of the Communist Party of China, State Council	http://www.gov.cn/gongbao/content/2008/content_912534.htm	4	3	3	3	1
47	2008/3/29	Notice	Notice of the issuance of the main points of work for 2008	State Council	http://www.gov.cn/zhengce/content/2008-04/16/content_1611.htm	4	3	3	1	5
48	2008/12/31	Opinion	Opinions on promoting stable agricultural development and sustainable income increase for farmers in 2009	Central Committee of the Communist Party of China, State Council	http://www.gov.cn/gongbao/content/2009/content_1220471.htm	4	3	3	1	1
49	2009/3/22	Opinion	Opinions on the sectoral division of labor for the implementation of the key work of the government work report	State Council	http://www.gov.cn/zhengce/content/2009-03/23/content_1531.htm	4	4	3	1	5
50	2009/5/10	Opinion	Opinions on the current stabilization of agricultural development and promotion of farmers' income increase	State Council	http://www.gov.cn/gongbao/content/2009/content_1322129.htm	4	4	4	1	5
51	2009/5/19	Notice	Notice on the issuance of the 2009 wheat minimum purchase price	Development and Reform Commission,	http://www.gov.cn/gongbao/content/2009/content_1417930.htm	2	4	4	3	5

			implementation plan	Ministry of Finance, Ministry of Agriculture, Grain Bureau, Agricultural Development Bank, China National Grain Storage Corporation						
52	2010/3/9	Opinion	Opinions on the integrated promotion of a new round of "grain basket" project construction	General Office of the State Council	http://www.gov.cn/zhengce/content/2010-03/12/content_2855.htm	3	4	4	3	1
53	2010/3/21	Opinion	Opinions on the sectoral division of labor for the implementation of the key work of the government work report	State Council	http://www.gov.cn/zhengce/content/2010-03/25/content_8210.htm	4	4	4	1	5
54	2011/3/19	Opinion	Opinions on the sectoral division of labor for the implementation of the key work of the government work report	State Council	http://www.gov.cn/zhengce/content/2011-03/23/content_1422.htm	4	4	4	1	5
55	2011/3/20	Opinion	Opinions on the implementation of the 2011 national action for stabilizing and increasing grain production	General Office of the State Council	http://www.gov.cn/gongbao/content/2011/content_1836363.htm	3	4	3	3	1
56	2011/12/31	Opinion	Opinions on accelerating agricultural science and technology innovation and continuously enhancing the ability to guarantee the	Central Committee of the Communist Party of China, State Council	http://www.gov.cn/gongbao/content/2012/content_2068256.htm	4	4	3	3	1

			supply of agricultural products							
57	2012/1/13	Notice	Notice on the issuance of the national plan for the development of modern agriculture (2011-2015)	State Council	http://www.gov.cn/zhengce/content/2012-02/13/content_2791.htm	4	4	4	1	1
58	2012/3/22	Opinion	Opinions on the sectoral division of labor for the implementation of the key work of the government work report	State Council	http://www.gov.cn/zhengce/content/2012-03/27/content_1197.htm	4	4	3	1	5
59	2012/12/31	Opinion	Opinions on accelerating the development of modern agriculture and further enhancing the vitality of rural development	Central Committee of the Communist Party of China, State Council	http://www.gov.cn/gongbao/content/2013/content_2332767.htm	4	4	4	3	1
60	2013/2/7	Opinion	Opinions on the implementation of the opinions of the State Council of the Central Committee of the Communist Party of China on accelerating the development of modern agriculture and further enhancing the dynamics of rural development	General Office of the State Council	http://www.gov.cn/zhengce/content/2013-02/16/content_2738.htm	3	2	2	1	5
61	2013/3/28	Opinion	Opinions on the division of labor among key departments for the implementation of the report	State Council	http://www.gov.cn/zhengce/content/2013-03/29/content_1045.htm	4	3	2	1	5

			on the work of the government and the spirit of the first plenary session of the State Council							
62	2014/2/23	Notice	Notice on the division of labor on the implementation of relevant policies and measures on several opinions of the Central Committee of the Communist Party of China and the State Council on comprehensively deepening rural reform and accelerating agricultural modernization	General Office of the State Council	http://www.gov.cn/zhengce/content/2014-03/10/content_8705.htm	3	3	3	3	5
63	2014/3/23	Opinion	Opinions on the sectoral division of labor for the implementation of the key work of the government work report	State Council	http://www.gov.cn/zhengce/content/2014-04/17/content_8766.htm	4	3	3	3	5
64	2014/8/10	Opinion	Opinions on accelerating the development of the modern insurance service industry	State Council	http://www.gov.cn/zhengce/content/2014-08/13/content_8977.htm	4	4	3	1	1
65	2014/11/20	Opinion	Opinions on guiding the orderly transfer of rural land management rights to develop adaptive scale agricultural management	General Office of the Central Committee, General Office of the State Council	http://www.gov.cn/gongbao/content/2014/content_2786719.htm	3	3	3	1	1
66	2014/12/31	Opinion	Opinions on the establishment of a sound responsibility system for food security governors	State Council	http://www.gov.cn/zhengce/content/2015-01/22/content_9422.htm	4	3	3	1	1
67	2015/2/1	Opinion	Several opinions on increasing reform and	Central Committee of the	http://www.gov.cn/gongbao/content/2015/cont	4	4	4	3	1

			innovation to accelerate agricultural modernization	Communist Party of China, State Council	ent_2818447.htm					
68	2015/3/25	Opinion	Opinions on the sectoral division of labor for the implementation of the key work of the government work report	State Council	http://www.gov.cn/zhengce/content/2015-04/10/content_9588.htm	4	3	3	3	5
69	2015/4/1	Notice	Notice on the issuance of guidance on leasing social grain storage facilities for collecting and storing state policy grain (for trial implementation)	Development and Reform Commission, Grain Bureau, Ministry of Finance, Agricultural Development Bank of China	http://www.gov.cn/gongbao/content/2015/content_2893174.htm	2	3	3	3	3
70	2015/11/2	Program	Comprehensive implementation program for deepening rural reform	General Office of the Central Committee, General Office of the State Council	http://www.gov.cn/gongbao/content/2015/content_2955704.htm	3	4	3	1	3
71	2015/11/3	Notice	Notice on the issuance of measures for the assessment of the responsibility system of governors for food security	General Office of the State Council	http://www.gov.cn/zhengce/content/2015-11/12/content_10286.htm	3	2	2	1	1
72	2015/12/31	Opinion	Opinions on implementing the new concept of development and accelerating the modernization of agriculture to achieve the goal of overall well-being	Central Committee of the Communist Party of China, State Council	http://www.gov.cn/gongbao/content/2016/content_5045927.htm	4	4	3	1	1

73	2016/6/23	Notice	Notice on the issuance of the measures for the administration of agricultural support protection subsidy funds	Ministry of Finance, Ministry of Agriculture	http://www.gov.cn/gongbao/content/2016/content_5139841.htm	2	4	3	1	3
74	2016/10/17	Notice	Notice on the issuance of a national plan for the modernization of agriculture (2016-2020)	State Council	http://www.gov.cn/zhengce/content/2016-10/20/content_5122217.htm	4	3	3	1	5
75	2016/11/24	Opinion	Opinions on improving support policies to promote sustainable income increase of farmers	General Office of the State Council	http://www.gov.cn/zhengce/content/2016-12/06/content_5143969.htm	3	3	3	1	5
76	2016/12/19	Notice	Notice on the issuance of the administrative measures for the subsidization of agricultural insurance premiums by the central finance government	Ministry of Finance	http://www.gov.cn/gongbao/content/2017/content_5217756.htm	2	4	4	3	3
77	2017/3/22	Opinion	Opinions on the sectoral division of labor for the implementation of the key work of the government work report	State Council	http://www.gov.cn/gongbao/content/2017/content_5186974.htm	4	4	3	1	5
78	2017/3/31	Opinion	Guiding Opinions on the establishment of functional grain production zones and important agricultural production protection zones	State Council	http://www.gov.cn/zhengce/content/2017-04/10/content_5184613.htm	4	3	3	1	1
79	2017/4/13	Opinion	Opinions on the key work of deepening economic system reform in 2017	State Council	http://www.gov.cn/gongbao/content/2017/content_5189007.htm	4	3	3	1	1
80	2017/4/28	Notice	Notice on the issuance of the measures for the	Ministry of Finance, Ministry	http://www.gov.cn/gongbao/content/2017/cont	2	3	3	3	1

			management of agricultural production development funds	of Agriculture	ent_5234539.htm					
81	2017/8/16	Opinion	Guiding opinions on accelerating the development of agricultural productive service industry	Ministry of Agriculture, Development and Reform Commission, Ministry of Finance	http://www.gov.cn/gongbao/content/2018/content_5271797.htm	2	3	3	1	1
82	2017/9/1	Opinion	Opinions on accelerating the structural reform of agricultural supply side and vigorously developing the grain industry economy	General Office of the State Council	http://www.gov.cn/zhengce/content/2017-09/08/content_5223640.htm	3	3	3	3	5
83	2017/9/30	Opinion	Opinions on innovative institutional mechanisms to promote green agricultural development	General Office of the Central Committee, General Office of the State Council	http://www.gov.cn/gongbao/content/2017/content_5232360.htm	3	4	3	1	1
84	2018/1/2	Opinion	Opinions on the implementation of the rural revitalization strategy	Central Committee of the Communist Party of China, State Council	http://www.gov.cn/gongbao/content/2018/content_5266232.htm	4	4	3	3	1
85	2018/2/22	Notice	Notice on the issuance of guidance on the implementation of agricultural machinery purchase subsidies for 2018-2020	Ministry of Agriculture and Rural Affairs, Ministry of Finance	http://www.gov.cn/zhengce/zhengceku/2018-12/31/content_5440951.htm	2	4	4	1	1
86	2018/4/1	Opinion	Opinions on the sectoral division of labor for the implementation of the key	State Council	http://www.gov.cn/zhengce/content/2018-04/12/content_5281920.	4	4	3	1	5

			work of the government work report		htm					
87	2018/5/4	Notice	Notice on the implementation of agricultural production development and other projects in 2018	Ministry of Agriculture and Rural Affairs, Ministry of Finance	http://www.gov.cn/zhengce/zhengceku/2018-12/31/content_5439236.htm	2	4	3	3	3
88	2018/7/2	Notice	Notice on the issuance of the technical guidelines for green agricultural development (2018-2030)	Ministry of Agriculture and Rural Development	http://www.gov.cn/gongbao/content/2018/content_5350058.htm	2	2	2	1	1
89	2018/7/6	Opinion	Guiding opinions on deepening cooperation in grain production and marketing to improve safety and security capacity	State Administration of Food and Material Reserves	http://www.gov.cn/zhengce/zhengceku/2018-12/31/content_5448288.htm	1	3	3	1	1
90	2018/7/30	Notice	Notice on matters relating to the inclusion of the three major grain crop seeds in the central government's agricultural insurance, insurance premium subsidy catalog	Ministry of Finance, Ministry of Agriculture and Rural Development, China Banking and Insurance Regulatory Commission	http://www.gov.cn/zhengce/zhengceku/2018-12/31/content_5441878.htm	2	4	4	1	3
91	2018/9/26	Plan	Strategic Plan for Rural Revitalization (2018-2022)	Central Committee of the Communist Party of China, State Council	http://www.gov.cn/gongbao/content/2018/content_5331958.htm	4	3	3	3	1
92	2018/12/21	Opinion	Guiding opinions on accelerating the transformation and upgrading of agricultural	State Council	http://www.gov.cn/gongbao/content/2019/content_5355467.htm	4	4	3	3	5

			mechanization and agricultural machinery and equipment industry							
93	2019/1/3	Opinion	The opinions on prioritizing agricultural and rural development and effectively carrying out the "Three Rural Issues" work	Central Committee of the Communist Party of China, State Council	http://www.gov.cn/gongbao/content/2019/content_5370837.htm	4	4	4	3	3
94	2019/1/21	Opinion	Opinions on the implementation of agricultural and rural work in 2019	Ministry of Agriculture and Rural Development	http://www.gov.cn/zhengce/zhengceku/2019-10/22/content_5443403.htm	2	3	3	3	1
95	2019/2/2	Notice	Notice on the issuance of the planting industry work points for 2019	Ministry of Agriculture and Rural Development	http://www.gov.cn/zhengce/zhengceku/2019-10/22/content_5443182.htm	2	4	3	1	3
96	2019/2/11	Notice	Notice on the issuance of the national strategic plan for quality agriculture (2018-2022)	Ministry of Agriculture and Rural Development, National Development and Reform Commission, Ministry of Science and Technology, Ministry of Finance, Ministry of Commerce, State Administration for Market Supervision and	https://www.moa.gov.cn/nybgb/2019/201902/201905/t20190517_6309469.htm	2	3	3	1	1

				Administration, State Administration of Grain and Material Reserves						
97	2019/2/15	Notice	Notice on the issuance of the main points of agricultural and rural science, education, environment and energy work in 2019	Ministry of Agriculture and Rural Development	http://www.gov.cn/zhengce/zhengceku/2019-10/22/content_5443214.htm	2	2	2	1	1
98	2019/2/27	Notice	Notice on unrelenting focus on grain production in 2019	Ministry of Agriculture and Rural Development	http://www.gov.cn/zhengce/zhengceku/2019-10/28/content_5445895.htm	2	3	4	3	3
99	2019/4/4	Notice	Notice on the implementation of agricultural production development and other programs in 2019	Ministry of Agriculture and Rural Development, Ministry of Finance	http://www.gov.cn/zhengce/zhengceku/2019-10/28/content_5446029.htm	2	3	3	1	1
100	2019/4/15	Opinion	Opinions on the establishment of a sound institutional mechanism and policy system for the integrated development of urban and rural areas	Central Committee of the Communist Party of China, State Council	http://www.gov.cn/gongbao/content/2019/content_5392288.htm	4	3	3	3	1
101	2019/4/19	Notice	Notice on accelerating the transformation and upgrading of agricultural mechanization	Ministry of Agriculture and Rural Development	http://www.gov.cn/zhengce/zhengceku/2019-10/28/content_5446035.htm	2	3	3	1	1
102	2019/6/17	Opinion	Opinion on promoting the revitalization of rural industries	State Council	http://www.gov.cn/zhengce/content/2019-06/28/content_5404170.htm	4	3	3	3	5

103	2019/8/23	Opinion	Guiding opinions on adhering to the goal of high-quality development and accelerating the construction of a modernized grain industry system	National Development and Reform Commission, National Bureau of Food and Material Reserves	http://www.gov.cn/zhengce/zhengceku/2019-08/30/content_5456448.htm	2	3	2	3	1
104	2019/9/19	Notice	Notice on the issuance of guidance on accelerating the high-quality development of agricultural insurance	Ministry of Finance, Ministry of Agriculture and Rural Development, State Forestry and Grassland Administration, China Banking and Insurance Regulatory Commission	http://www.gov.cn/zhengce/zhengceku/2019-12/03/content_5458076.htm	2	3	3	1	3
105	2019/11/13	Opinion	Opinions on effectively strengthening the construction of high-standard farmland to enhance the ability to guarantee national grain security	General Office of the State Council	http://www.gov.cn/zhengce/content/2019-11/21/content_5454205.htm	3	4	3	5	5
106	2020/1/2	Opinion	Opinions on grasping the key work in the field of "Three Rural Issues" to ensure the realization of comprehensive well-being on schedule	Central Committee of the Communist Party of China, State Council	http://www.gov.cn/gongbao/content/2020/content_5480477.htm	4	3	3	1	1

107	2020/2/10	Opinion	Opinions on the implementation of the Party Central Committee and the State Council on the implementation of the deployment of key agricultural and rural work in 2020	Ministry of Agriculture and Rural Development	http://www.gov.cn/zhengce/zhengceku/2020-02/13/content_5478017.htm	2	3	3	3	1
108	2020/4/14	Notice	Notice on the implementation of agricultural production development and other programs in 2020	Ministry of Finance, Ministry of Agriculture and Rural Development	http://www.gov.cn/zhengce/zhengceku/2020-04/20/content_5504504.htm	2	4	4	5	3
109	2020/4/25	Notice	Notice on the conscientious implementation of the food security governor's responsibility system in 2020	Ministry of Natural Resources, Ministry of Agriculture and Rural Development, National Development and Reform Commission, Ministry of Finance, Ministry of Ecology and Environment, Ministry of Water Resources, General Administration of Customs, State Administration	http://www.gov.cn/zhengce/zhengceku/2020-05/15/content_5511922.htm	2	3	3	3	1

				for Market Supervision and Administration, National Bureau of Statistics, State Administration of Food and Material Reserves						
110	2020/6/6	Opinion	Opinions on the sectoral division of labor for the implementation of the key work of the government work report	State Council	http://www.gov.cn/zhengce/content/2020-06/11/content_5518699.htm	4	3	3	1	3
111	2020/11/4	Opinion	Opinions on preventing "non-grain conversion" of cultivated land and stabilizing grain production	General Office of the State Council	http://www.gov.cn/zhengce/content/2020-11/17/content_5562053.htm	3	4	4	3	5
112	2021/1/4	Opinion	Opinions on comprehensively promoting rural revitalization and accelerating agricultural and rural modernization	Central Committee of the Communist Party of China, State Council	http://www.gov.cn/gongbao/content/2021/content_5591401.htm	4	3	3	3	1
113	2021/1/8	Opinion	On the implementation of the Party Central Committee, the State Council 2021 key work in agriculture and rural areas to implement the deployment of opinions	Ministry of Agriculture and Rural Development	http://www.gov.cn/zhengce/zhengceku/2021-03/01/content_5589458.htm	2	4	3	3	1
114	2021/2/15	Regulation	Regulations on the management of grain circulation	State Council	https://www.gov.cn/zhengce/content/202211/content_6611685.htm	4	4	3	3	1
115	2021/3/12	Notice	Notice on the issuance of guidance on the implementation of	Ministry of Finance, Ministry of Agriculture and	http://www.gov.cn/zhengce/zhengceku/2021-04/07/content_5598134.	2	3	3	1	1

			agricultural machinery purchase subsidies for 2021-2023	Rural Development	htm					
116	2021/3/19	Opinion	Opinions on the sectoral division of labor for the implementation of the key work of the government work report	State Council	http://www.gov.cn/gongbao/content/2021/content_5598115.htm	4	3	3	3	5
117	2021/4/20	Opinion	Opinions on comprehensively promoting the rule of law in agriculture and rural areas	Ministry of Agriculture and Rural Development	http://www.gov.cn/zhengce/zhengceku/2021-04/28/content_5603529.htm	2	3	3	3	1
118	2021/4/30	Notice	Notice on the implementation of agricultural production development and other projects in 2021	Ministry of Finance, Ministry of Agriculture and Rural Development	http://www.gov.cn/zhengce/zhengceku/2021-05/14/content_5606462.htm	2	3	3	1	1
119	2021/6/18	Opinion	Opinions on further promoting the quality grain project	Ministry of Finance, State Administration of Food and Material Reserves	http://www.gov.cn/zhengce/zhengceku/2021-06/29/content_5621465.htm	2	3	3	1	1
120	2021/6/24	Notice	Notice on the expansion of the implementation scope of full cost insurance and planting income insurance for three major grain crops	Ministry of Finance, Ministry of Agriculture and Rural Development, China Banking and Insurance Regulatory Commission	http://www.gov.cn/zhengce/zhengceku/2021-06/29/content_5621466.htm	2	4	3	3	3

121	2021/7/2	Regulation	Regulations on the implementation of the land administration law of the People's Republic of China	State Council	http://www.gov.cn/zhengce/content/2021-07/30/content_5628461.htm	4	4	4	5	3
122	2021/9/2	Notice	Notice on constant attention to machine harvesting and loss reduction as the main work of mechanization of grain production	Ministry of Agriculture and Rural Development	http://www.gov.cn/zhengce/zhengceku/2021-09/07/content_5635835.htm	2	3	3	3	1
123	2021/10/31	Program	Grain conservation action program	General Office of the Central Committee, General Office of the State Council	http://www.gov.cn/gongbao/content/2021/content_5651724.htm	3	4	3	3	1
124	2021/11/12	Notice	Notice on the issuance of the fourteenth five-year plan for the modernization of agriculture and rural areas	State Council	http://www.gov.cn/zhengce/content/2022-02/11/content_5673082.htm	4	4	3	1	1
125	2021/11/13	Notice	Notice on the issuance of the program of the "six enhancement actions" of the quality grain project	State Administration of Food and Material Reserves	http://www.gov.cn/zhengce/zhengceku/2021-11/18/content_5651565.htm	1	3	2	1	1
126	2021/11/17	Opinion	Guiding opinions on expanding the multiple functions of agriculture and promoting the high-quality development of rural industries	Ministry of Agriculture and Rural Development	http://www.gov.cn/zhengce/zhengceku/2021-11/19/content_5651881.htm	2	4	4	3	1
127	2021/11/27	Notice	Notice on issues related to the strict control of the use of cultivated land	Ministry of Natural Resources,	http://www.gov.cn/zhengce/zhengceku/2021-12/26/content_5664643.	2	4	4	5	1

				Ministry of Agriculture and Rural Development, State Forestry and Grassland Administration	htm					
128	2021/12/31	Notice	Circular on the issuance of the administrative measures on premium subsidies for agricultural insurance of the central government	Ministry of Finance	http://www.gov.cn/gongbao/content/2022/content_5683856.htm	2	4	3	1	3
129	2022/1/4	Opinion	Opinions on Completing the Key Work of Comprehensively Promoting Rural Revitalization in 2022	Central Committee of the Communist Party of China, State Council	http://www.gov.cn/gongbao/content/2022/content_5678065.htm	4	4	3	3	1
130	2022/1/14	Opinion	Implementation opinions on implementing the deployment of key work on comprehensively promoting rural revitalization in 2022 by the party central committee and state council	Ministry of Agriculture and Rural Development	http://www.gov.cn/zhengce/zhengceku/2022-03/01/content_5676262.htm	2	4	4	3	3
131	2022/3/10	Notice	Notice on further improving the work related to the production of grain, soybeans and other important agricultural products	National Development and Reform Commission	http://www.gov.cn/zhengce/zhengceku/2022-03/19/content_5679800.htm	2	4	4	3	1
132	2022/3/14	Notice	Notice on the promotion of mechanized rice planting in South China	Ministry of Agriculture and Rural Development	http://www.gov.cn/zhengce/zhengceku/2022-03/21/content_5680206.htm	2	3	3	3	1

133	2022/3/21	Opinion	Opinions on the division of key work in the implementation of the Government's Work Report	State Council	http://www.gov.cn/zhengce/content/2022-03/25/content_5681343.htm	4	4	3	3	5
134	2022/5/9	Notice	Notice on the implementation of projects such as agricultural production development in 2022	Ministry of Finance, Ministry of Agriculture and Rural Development	http://www.gov.cn/zhengce/zhengceku/2022-05/13/content_5690136.htm	2	3	3	3	1
135	2022/5/24	Notice	Notice on the issuance of a package of policy measures to stabilize the economy in a solid manner	State Council	http://www.gov.cn/zhengce/content/2022-05/31/content_5693159.htm	4	3	3	1	1
136	2023/1/2	Opinion	Opinions on completing the key work of comprehensively promoting rural revitalization in 2023	Central Committee of the Communist Party of China, State Council	http://www.gov.cn/gongbao/content/2023/content_5743582.htm	4	4	3	3	1
137	2023/2/3	opinion	Implementation opinions on implementing the deployment of key work on comprehensively promoting rural revitalization in 2023 by the party central committee and state council	Ministry of Agriculture and Rural Development	http://www.gov.cn/zhengce/zhengceku/2023-02/22/content_5742671.htm	2	4	4	3	3
138	2023/2/10	Notice	Notice on further improving the quality of mechanized sowing	Ministry of Agriculture and Rural Development	http://www.gov.cn/zhengce/zhengceku/2023-02/15/content_5741576.htm	2	3	3	3	3
139	2023/4/7	Notice	Notice on the issuance of the measures for the management of funds for the construction and utilization of cultivated land	Ministry of Finance, Ministry of Agriculture and Rural Development	https://www.gov.cn/gongbao/2023/issue_10566/202307/content_6890782.html	2	3	3	3	1

140	2023/5/9	Opinion	Opinions on accelerating the construction of drying capacity in grain producing areas	National Development and Reform Commission, Ministry of Finance, Ministry of Natural Resources, Ministry of Ecology and Environment, Ministry of Agriculture and Rural Affairs, State Administration of Food and Material Reserves	https://www.gov.cn/zhengce/zhengceku/202305/content_6857350.htm	2	4	3	3	3
141	2023/7/7	Notice	Notice on expanding the implementation scope of full cost Insurance and planting income insurance for the three major grain crops to all large grain producing counties in the country	Ministry of Finance, Ministry of Agriculture and Rural Development, State Administration of Financial Supervision and Administration	https://www.gov.cn/zhengce/zhengceku/202307/content_6891795.htm	2	3	3	1	3
142	2023/7/28	Measure	Measures for the regulation of grain quality and safety	National Development and Reform Commission	https://www.gov.cn/gongbao/2023/issue_10726/202309/content_6906518.html	2	3	4	3	3

143	2023/9/12	Notice	Notice on the acquisition of autumn grain in 2023	National Development and Reform Commission, State Administration of Grain and Material Reserves, Ministry of Finance, Ministry of Transportation, Ministry of Agriculture and Rural Development, People's Bank of China, State Administration for Market Supervision and Regulation, State Administration of Financial Supervision and Regulation, China State Railway Group Corporation Ltd.	https://www.gov.cn/zhengce/zhengceku/202309/content_6904344.htm	2	4	4	3	3
144	2023/9/25	Opinion	Implementation opinions on promoting the high-quality development of inclusive finance	State Council	https://www.gov.cn/gongbao/2023/issue_10786/202310/content_6912660.html	4	4	3	3	1

145	2023/12/29	Law	Law of the people's republic of China on food security	Central Committee of the Communist Party of China	https://www.gov.cn/yaowen/liebiao/202312/content_6923387.htm	4	3	3	1	1
